# Molecular Characteristics of Community-Associated *Staphylococcus aureus* Isolates From Pediatric Patients With Bloodstream Infections Between 2012 and 2017 in Shanghai, China

**DOI:** 10.3389/fmicb.2018.01211

**Published:** 2018-06-06

**Authors:** Xing Wang, Qian Liu, He Zhang, Xia Li, Weichun Huang, Qihua Fu, Min Li

**Affiliations:** ^1^Department of Laboratory Medicine, Shanghai Children’s Medical Center, Shanghai Jiao Tong University School of Medicine, Shanghai, China; ^2^Department of Laboratory Medicine, Renji Hospital, Shanghai Jiao Tong University School of Medicine, Shanghai, China; ^3^Department of Emergency, Hebei Provincial Hospital of Traditional Chinese Medicine, Hebei, China; ^4^Department of Medical Microbiology and Immunology, University of California, Davis, Davis, CA, United States

**Keywords:** bloodstream infections, methicillin-resistance *S. aureus*, methicillin-susceptible *S. aureus*, multidrug resistance, sequence typing, virulence genes

## Abstract

*Staphylococcus aureus* is known as an invasive human pathogen, resulting in significant morbidity and mortality worldwide; however, information on community-associated *S. aureus* (CA-SA) from bloodstream infections (BSI) in children in China remains scarce. This study aimed to investigate the molecular characteristics of 78 CA-SA isolates recovered from pediatric patients with BSI between 2012 and 2017 in Shanghai. All isolates including 51 (65.4%) methicillin-susceptible *S. aureus* (MSSA) and 27 (34.6%) methicillin-resistant *S. aureus* (MRSA) isolates were characterized based on antimicrobial resistance, virulence genes, multilocus sequence typing (MLST), *spa*, and SCC*mec* typing. A total of 18 distinct sequence types (STs) and 44 *spa* types were identified. ST188 and ST7 were the predominant MSSA clones and ST59-MRSA-SCC*mec*IV/V was the most common MRSA clone. *Spa* t189 (9.0%, 7/78) was the most common *spa* type. SCC*mec* types IV and V were observed at frequencies of 59.3 and 40.7%, respectively. Notably, 40 (51.3%) *S. aureus* BSI strains were multidrug resistant (MDR), and these were mostly resistant to penicillin, erythromycin, and clindamycin. MRSA strains were associated with substantially higher rates of resistance to multiple antibiotics than MSSA strains. Fifty (64.1%, 50/78) isolates, including 19 (70.3%) MRSA isolates, harbored ≥ 10 tested virulence genes, as evaluated in this study. Ten (37.0%) MRSA isolates and four (7.8%) MSSA isolates harbored the gene encoding Panton–Valentine leukocidin (PVL). Virulence genes analysis showed diversity in different clones; the *seb*-*sek*-*seq* genes were present in all ST59 strains, whereas the *seg*-*sei*-*sem*-*sen*-*seo* genes were present in different clones including ST5, ST20, ST22, ST25, ST26, ST30, ST121, and ST487 strains. In conclusion, this study revealed that community-associated *S. aureus* strains from BSI in children demonstrated considerable genetic diversity, and identified major genotypes of CA-MRSA and CA-MSSA, with a high prevalence of CA-MRSA. Furthermore, major genotypes were frequently associated with specific antimicrobial resistance and toxin gene profiles. Understanding the molecular characteristics of those strains might provide further insights regarding the spread of BSI *S. aureus* among children between communities in China.

## Introduction

*Staphylococcus aureus* can cause a wide variety of diseases ranging from mild skin and soft-tissue infections to severe systemic infections in human and animals (Lowy, 1998). Serious *S. aureus* infections such as bacteremia are generally associated with high morbidity and mortality, and the acquisition of methicillin resistance further limits therapeutic options (Cosgrove et al., 2003; Wang et al., 2008; Bassetti et al., 2012). Since methicillin-resistant *S aureus* was first identified at a hospital in the United Kingdom in 1961, it quickly became an important pathogen globally (referred to as hospital-associated MRSA; HA-MRSA). Until the late 1980s and early 1990s, some cases of MRSA in young and otherwise healthy patients without hospital-related risk factors were reported. These isolates were called community-associated MRSA (CA-MRSA) and their emergence and spread increased the risk to public health.

Compared to traditional HA-MRSA strains, CA-MRSA isolates harbor different types of SCC*mec* elements encoding methicillin(*mec*) resistance genes. To date, 13 SCC*mec* types (indicated by roman numerals I to XIII) and three *mec*(*mec*A/B/C) genes have been identified among *S. aureus* in the world (Hiramatsu et al., 2013; Kaya et al., 2018). The majority of CA-MRSA isolates have SCC*mec* type IV or V, do not exhibit resistance to multiple antibiotics (except to β-lactams), and possess different exotoxin gene profiles (Dinges et al., 2000). Within the last decade, rates of CA-MRSA infection have been increasing, while HA-MRSA infection rates have generally declined (David et al., 2014). The epidemiological impact of CA-MRSA strains is believed to stem from a combination of methicillin resistance and extraordinary virulence, allowing these strains to infect otherwise healthy individuals and spread rapidly throughout the population. As a result, there is an urgent need to understand the molecular characteristics of CA-MRSA isolates to achieve more effective infection control.

Community-associated-Methicillin-resistant *S. aureus* strains are considered more virulent than HA-MRSA strains because they possess specific virulence factors. The increased expression of various genes has also been associated with increased virulence in CA-MRSA (Otto, 2013). For example, Panton–Valentine leukocidin (*pvl*), a bicomponent leukotoxin virulence factor, has been linked to severe skin and soft tissue infections and necrotizing pneumonia caused by CA-MRSA strains (Lina et al., 1999), but has also been found in HA-MRSA and CA-MSSA isolates. The superantigen exotoxin (TSST-1), exfoliatin A (ETA) and B (ETB), staphylococcal enterotoxins (SEs) found to be closely related to different types of staphylococcal infections (Hanakawa et al., 2002; Jarraud et al., 2002; Warner and Onderdonk, 2004). However, the significance of these factors in CA-SA bacteremia is not well understood.

It has been found that various CA-SA clones, and especially CA-MRSA, circulate in different countries or regions. For example, ST1-IV and ST8-IV clones are mainly found in the United States and Canada, whereas ST80-IV clones are more prevalent in Europe and ST59-IV/V are the most common CA-MRSA clones in China and several other Asian countries. The prevalence of CA-SA varies with age, geography, disease, and time, and the occurrence rate of CA-MRSA varies substantially worldwide, ranging from < 1% to > 50% in different countries (Deurenberg and Stobberingh, 2008; Chen and Huang, 2014). The incidence of CA-SA infections is higher in children (Huang and Chen, 2011). Although CA-SA infection and transmission has become a serious public health problem worldwide (David et al., 2015; McMullan et al., 2016; Pena Amaya et al., 2017; Kang et al., 2017; Roediger et al., 2017), the information regarding CA-SA associated with bloodstream infections (BSIs) in children in China is still very limited. The aim of this study was to investigate the molecular profile, antimicrobial resistance, and virulence genes associated with 78 CA-SA isolates recovered from pediatric patients with BSIs between 2012 and 2017 in a hospital in China.

## Materials and Methods

### Bacterial Isolates

From July 2012 to February 2017, 78 non-duplicate community-associated *S. aureus* isolates were collected from pediatric patients (< 18 years old) with BSIs in a university hospital (Shanghai Children’s Medical Center) in Shanghai. Shanghai Children’s Medical Center (SCMC), affiliated with Shanghai Jiao Tong University, is one of the largest pediatric hospitals in China with 604 beds and approximately 6,000 hospital admissions per day.

*Staphylococcus aureus* isolates were confirmed by classic microbiological methods including Gram stain and catalase and coagulase activity with rabbit plasma. They were further identified by biochemical characterization using the API-Staph test (bioMérieux, Lyon, France). MRSA isolates were initially identified using cefoxitin screening and the presence of the *mecA* gene was confirmed by PCR (Kondo et al., 2007). CA-MRSA was defined as an MRSA isolate that was obtained either from an outpatient or an inpatient within 48 h of hospitalization, and without the patient having a medical history of MRSA infection or colonization, admission to a healthcare facility, dialysis, surgery or the insertion of indwelling devices in the past year (Skov et al., 2012). These isolates were processed in Class II Biological Safety Cabinets. All strains were stored at -70°C and grown overnight on sheep blood agar plates at 37°C.

This study was approved by the Ethics Committee of SCMC, and the Review Board exempted the need for written informed consent because it mainly focused on bacteria and patient intervention did not occur.

### Antimicrobial Susceptibility Testing

The antibiotic susceptibility profiles of all *S. aureus* isolates in the current study were performed using the bioMérieux VITEK2 system following manufacturer’s instructions. Results were interpreted according to the recommendations and definitions of the Clinical and Laboratory Standards Institute [CLSI] (2015). The following 16 drugs were tested: cefoxitin (FOX), linezolid (LZD), ciprofloxacin (CIP), clindamycin (DA), erythromycin (E), trimethoprim-sulfamethoxazole (SXT), moxifloxacin (MOF), vancomycin (V), tetracycline (TET), penicillin (P), rifampicin (RF), levofloxacin (LVX), oxacillin (OXA), gentamicin (GM), quinupristin/dalfopristin (Q/D), and tigecycline (TGC). *S. aureus* ATCC 29213 was used for quality control.

### Multilocus Sequence Typing (MLST) Analysis

All *S. aureus* isolates were screened according to the protocol described on the *S. aureus* MLST website^1^ (Enright and Spratt, 1999; Aanensen and Spratt, 2005). PCR amplicons of seven *S. aureus* housekeeping genes were obtained from chromosomal DNA. DNA was extracted as previously described (Hartmann et al., 1997). The sequences of the PCR products were compared to those of the existing alleles available from the MLST website, and the allelic number (sequence type, ST) was determined for each sequence. Clustering of related STs that were defined as cloned complexes (CCs) was performed using the eBURST (Based Upon Related Sequence types) algorithm.

### SCC*mec* Typing

Methicillin-resistant *S. aureus* isolates were subjected to SCC*mec* typing as described by Kondo et al. (2007), which is based on a set of multiplex PCR reactions with 14 primers. SCC*mec* types I–V were assigned according to the combination of the cassette chromosome recombinase (*ccr*) type and *mec* class. MRSA isolates that could not be assigned to any expected type were defined as non-typable (NT).

### *spa* Typing

In *S. aureus*, the polymorphic X region of the staphylococcal protein A-encoding (*spa)* gene was amplified and sequenced as described previously (Shopsin et al., 1999; Koreen et al., 2004). *spa* typing was assigned by submitting the data to the *S. aureus* *spa* type database^2^.

### Detection of Virulence Genes

All *S. aureus* isolates were screened for the following 33 staphylococcal virulence genes: staphylococcal enterotoxin genes (*sea*, *seb*, *sec, sed, see, seg, seh, sei*, *sej*, *sel*, *sem*, *sen*, *seo*, *sep*, *seq*, *sek*), toxic shock syndrome toxin (*tsst1*), arginine catabolic mobile gene (*arcA*), exfoliative toxin genes (*eta*, *etb*), leukocidin (*luk*F/S-PV, *luk*E, *luk*M) (Lina et al., 1999), bacteriocin (*bsaA*), hemolysin genes (*hla*, *hlb*, *hlg, hlg2*), and adhesin genes (*clfA*, *icaA*, *sdrC*, *sdrD*, and *sdrE*), as previously described (Arvidson and Tegmark, 2001; Peacock et al., 2002; Wardenburg et al., 2007).

The amplification was carried out on a GeneAmp 9700 thermal cycler (Applied Biosystems, NY, United States) under the following conditions: an initial 5 min denaturation at 94°C, followed by 35 cycles of 30 s at 94°C, 30 s at 55°C, and 30 s at 72°C, with a final extension at 72°C for 7 min. In each PCR, *S. aureus* isolates harboring virulence genes determined by our previous study were used as positive control strains (Wang et al., 2016) and distilled water was used for a negative control. The PCR fragments were visualized by agarose gel electrophoresis and ethidium bromide staining.

### Statistical Analysis

Statistical analyses were performed using Stata software (version 10.1/SE, Stata Corp, College Station, TX, United States), using χ2 and Fisher’s exact tests, as appropriate for the analysis of categorical data. Statistical significance was set at *P* ≤ 0.05.

## Results

### Prevalence of CA-SA From Pediatric Patients With BSIs

From blood culture samples during 2012–2017, we found positive rate of CA-SA to be 0.2076% (13/6261*)* in 2013, 0.1223% (10/8174) in 2014, 0.1696% (15/8845) in 2015, and 0.2135% (21/9834) in 2016 among pediatric patients. Laboratory-based surveillance indicated a relatively stable number of CA-SA BSI infections in our hospital, with the number of cases increasing from 2014 to 2016 over time.

### MLST, SCC*mec*, and *spa* Typing

The evolutionary and genetic diversity of 78 *S. aureus* isolates from BSIs in children was analyzed by MLST (**Table 1**). There were 18 distinct STs identified within the 78 isolates, of which the most frequently represented were ST59 (15%, 12/78) and ST188 (15%, 12/78), accounting for nearly one third of all *S. aureus* isolates, followed by ST7 (14%, 11/78), ST398 (12%, 9/78), ST88 (7.7%, 6/78), ST5 (7.7%, 6/78), ST6 (6.4%, 5/78), and ST1 (5.1%, 4/78). Other STs accounted for one or two isolates, respectively.

**Table 1:** Molecular characteristics and antibiotic resistance profiles of 78 BSI isolates from pediatric patients.

	CCs	MLST(n,%)	*spa Type(n,%)*	SCC*mec* type	Antimicrobial resistance(R%)
MRSA	CC59	ST59(12,15.4%)	t172(4,5.1%),t437(3,3.8%), t441(3,3.8%),t1751(1,1.3%),t3485(1,1.3%)	IV(8,10.3%),V(4,5.1%)	FOX(100),DA(83.3),E(83.3),TET(50),P(100), OXA(100)
	CC398	ST398(5,6.4%)	t034(3,3.8%),t1446(1,1.3%),NT(1,1.3%)	IV(1,1.3%),V(4,5.1%)	FOX(100),DA(40),E(40),TET(25),P(100), OXA(100)
	CC88	ST88(3,3.8%)	t2310(1,1.3%),t12147(1,1.3%),NT(1,1.3%)	IV(1,1.3%),V(2,2.6%)	FOX(100),DA(66.7),E(66.7),SXT(33.3), P(100),LEV(33.3),OXA(100)
	CC1	ST1(2,2.6%)	t114(2,2.6%)	IV(2,2.6%)	FOX(100),DA(50),E(50),P(100),OXA(100)
	CC188	ST188((1,1.3%)	t2769(1,1.3%),	IV(1,1.3%)	FOX(100),P(100),OXA(100)
	CC25	ST25(1,1.3%)	t349(1,1.3%)	IV(1,1.3%)	FOX(100),DA(100),E(100),P(100),OXA(100)
	CC30	ST30(1,1.3%)	t019(1,1.3%)	IV(1,1.3%)	FOX(100),P(100),OXA(100)
	CC8	ST630(1,1.3%)	t4549(1,1.3%)	V(1,1.3%)	FOX(100),CIP(100),DA(100),E(100), MOF(100),TET(100), P(100),RD(100),LEV(100),OXA(100), GM(100)
	CC72	ST72(1,1.3%)	t148(1,1.3%)	IV(1,1.3%)	FOX(100),P(100),OXA(100)
MSSA	CC7	ST7(11,14.1%)	t091(6,7.7%),t605(1,1.3%),t803(1,1.3%), t14204(1,1.3%),NT(2,2.6%)		CIP(9.1),DA(27.3),E(27.3),TET(63.6),P(90.9)
	CC188	ST188(11,14.1%)	t189(7,9.0%),t5229(2,2.6%),t2769(1,1.3%), t7290(1,1.3%)		CIP(9.1),DA(18.2),E(27.3),TET(9.1),P(90.9)
	CC5	ST5(6,7.7%)	t548(3,3.8%),t062(1,1.3%),t2764(1,1.3%), t4336(1,1.3%)		DA(33.3),E(33.3),SXT(16.7),P(100), GM(16.7)
		ST487((1,1.3%)	t442((1,1.3%)		
	CC6	ST6(5,6.4%)	t701(4,5.1%),t2467(1,1.3%)		DA(40),E(40),P(80)
	CC398	ST398(4,5.1%)	t034(1,1.3%),t571(1,1.3%),t1456(1,1.3%), t1580(1,1.3%)		CIP(25),DA(50),E(50),SXT(25),TET(25),P(75)
	CC88	ST88(3,3.8%)	t1376(1,1.3%),t2592(1,1.3%),t12858(1,1.3%)		DA(33.3),E(33.3),TET(33.3),P(100)
	CC1	ST1(2,2.6%)	t127(1,1.3%),t14384(1,1.3%)		DA(50),E(50),SXT(100),P(100),GM(50)
	CC121	ST121(2,2.6%)	t2019(1,1.3%),t2091(1,1.3%)		DA(100),E(100),SXT(50),P(100)
	CC20	ST20(2,2.6%)	t164(2,2.6%)		DA(50),E(50),P(100)
	CC15	ST15(1,1.3%)	t328(1,1.3%)		DA(100),E(100),P(100)
	CC30	ST30(1,1.3%)	t338(1,1.3%)		DA(100),E(100),P(100)
	CC22	ST22(1,1.3%)	t309(1,1.3%)		P(100)
	CC25	ST26(1,1.3%)	t078(1,1.3%)		SXT(100),P(100)

The eBURST analysis of *S. aureus* using all STs available in the MLST database is shown. These strains were clustered by eBURST into 16 CCs (CC59, CC188, CC7, CC398, CC88, CC5, CC6, CC1, CC20, CC30, CC121, CC25, CC72, CC8, CC15, and CC22). The largest clusters were CC59 and CC188 each with 12 isolates, followed by CC7 with 11 isolates, CC398 with nine isolates, CC5 with seven isolates, CC88 with six isolates, CC6 with five isolates and CC1 with four isolates. The remaining CCs harbored one or two strains (Supplementary Table S1).

The genetic diversity of these isolates was confirmed by *spa* typing. Forty-four *spa* types were observed, *spa* t189 (9.0%, 7/78) was the most predominant type, followed by t091(7.7%, 6/78), t172(5.1%, 4/78), t034(5.1%, 4/78), t701(5.1%, 4/78), t437(3.8%, 3/78), and t441(3.8%, 3/78). Each of the remaining *spa* types was represented by fewer than three isolates (**Table 1**).

By SCC*mec* typing, only two types (types IV and V) were found among 27 MRSA isolates. The most common was type IV, which comprised 16 isolates (59.3%, 16/27), whereas type V comprised 11 isolates (40.7%, 11/27; **Table 1**).

### Antimicrobial Susceptibility Testing

The antimicrobial resistance profiles of 78 *S. aureus* BSI isolates according to MLST are listed in **Table 1**. All the strains were susceptible to linezolid, vancomycin, quinupristin/dalfopristin, and tigecycline. The majority were resistant to penicillin (93.6%), erythromycin (46.2%), and clindamycin (44.9%); however, they were susceptible to most of the antibiotics tested. The resistance rates to other antibiotics tested were 34.6% to cefoxitin or oxacillin, 23.1% to tetracycline, 9% to trimethoprim-sulfamethoxazole, 5.1% to fluoroquinolones (5.1% to ciprofloxacin, 2.6% to levofloxacin, 1.3% to moxifloxacin), 3.8% to gentamicin, and 1.3% to rifampicin (Supplementary Table S2).

Among the 78 S. *aureus* isolates, 40 (51.3%) strains were resistant to ≥ 3 distinct classes of antibiotics, including 23 (29.5%) MRSA and 17 (21.8%) MSSA strains. Twenty (25.6%) isolates were resistant to five or more antibiotics, six (7.7%) were resistant to four antibiotics, and eighteen (23.1%) were resistant to three antibiotics. Among MSSA strains, 17 (21.8%) were resistant to ≥ 3 antibiotics, eight (10.3%) showed resistance to ≥ 4 antibiotics, and four (5.1%) were resistant to ≥ 5 antibiotics, However, all MRSA strains were found to be resistant to at least three tested antibiotics.

### Virulence Gene Profiles

The distribution of 33 putative virulence genes varied among the 78 BSI strains according to ST (**Table 2**). All of these virulence genes except *lukM*, *etb*, and *arcA* genes were identified within multiple isolates, and all isolates simultaneously harbored at least six virulence genes. Fifty (64.1%, 50/78) isolates harbored ≥ 10 tested virulence genes, among which one isolate harbored 17 genes, five isolates had 15 genes, seven isolates contained 14 genes, 14 isolates harbored 13 genes, eight isolates had 12 genes, 10 isolates contained 11 genes, and five isolates harbored 10 genes.

**Table 2:** Frequencies of virulence and enterotoxin genes among the molecular types of 78 bloodstream infection *Staphylococcus aureus* isolates from pediatric patients.

	CCs	Virulence genes detected	Enterotoxin genes detected
MRSA	CC59	*pvl(41.7),hla(100),hlb(100),hlg2(91.7),bsa(8.3),icaA(100),clfA(100),sdrC(100), sdrD(16.7),sdrE(100),lukE(66.7)*	*sea(50),seb(100),sec(16.7),sed(8.3),see(8.3), seq(100),sek(100),sep(8.3)*
	CC398	*pvl(20),hla(100),hlg(100),hlg2(40),bsa(60),icaA(100),clfA(100),sdrC(100),sdrE(40), lukE(60)*	
	CC88	*pvl(66.7),hla(100),hlb(66.7),hlg2(100),icaA((100),clfA(100),sdrC(100),sdrD(66.7), sdrE(66.7),lukE(100)*	*seb(33.3),sec(33.3),sed(33.3),see(100),sep(100)*
	CC1	*hla(100),hlb(100),hlg2(50),icaA((100),clfA(100),sdrC(100),sdrD(100),sdrE(100), bsa(50), lukE(100),tsst(100)*	*sec(100),seh(50),sek(100),seq(100),sel(100)*
	CC188	*hla(100),hlb(100),hlg2(100),icaA((100),clfA(100),sdrC(100),sdrE(100),lukE(100)*	*sea(100),seb(100),sec(100),sed(100),seq(100),sek(100)*
	CC25	*pvl(100),hla(100),hlb(100),hlg2(100),icaA((100),clfA(100),sdrC(100), sdrD(100),sdrE(100),bsa(100),lukE(100)*	*seb(100),seg(100),sei(100),sem(100),sen(100),seo(100)*
	CC30	*pvl(100),hla(100),hlg(100),hlg2(100),icaA((100),clfA(100),sdrC(100),lukE(100)*	*seg(100),sei(100),sem(100),sen(100),seo(100)*
	CC8	*hla(100),hlb(100),hlg2(100),icaA((100),clfA(100),sdrC(100),lukE(100)*	
	CC72	*hla(100),hlg2(100),icaA((100),clfA(100),sdrC(100),sdrD(100),lukE(100)*	*sei(100),sem(100),sen(100),seo(100)*
MSSA	CC7	*hla(100),hlb(54.5),hlg2(100),icaA((100),clfA(100),sdrC(100),sdrD(100), sdrE(27.3),bsa(18.2),lukE(90.9)*	*sea(9.1),sec(36.4),sed(9.1),see(81.8),seh(9.1), sei(9.1),sep(81.8)*
	CC188	*hla(100),hlb(9.1),hlg2(100),icaA(100),clfA(100),sdrC(100),sdrE(100),lukE(100)*	*seb(18.2)*
	CC5	*hla(100),hlb(42.9),hlg2(85.7),icaA((100),clfA(100),sdrD(57.1),sdrE(100),lukE(100)*	*sea(28.6),sec(14.3),sed(71.4),seg(100),sei(100), sej(71.4),sel(14.3),sem(100),sen(100),seo(100)*
	CC6	*hla(100),hlb(20),hlg2(100),icaA((100),clfA(100),sdrC(100),sdrD(100), sdrE(100),bsa(100),lukE(100),eta(40)*	*sea(100),sec(20),sed(20)*
	CC398	*pvl(25),hla(100),hlb(25),hlg(100),icaA(100),clfA(100),sdrC(75),sdrE(75),lukE(75)*	
	CC88	*pvl(33.3),hla(100),hlb(66.7),hlg2(100),icaA(100),clfA(100),sdrC(100), sdrE(33.3),bsa(33.3),lukE(100)*	*see(100),sep(100)*
	CC1	*hla(100),hlb(100),hlg2(100),icaA(100),clfA(100),sdrC(100),sdrD(100), sdrE(100),bsa(50),lukE(100)*	*sea(50),sec(100),seh(100),sel(50)*
	CC121	*hla(100),hlb(50),hlg2(100),icaA((100),clfA(100),lukE(100),eta(50)*	*seg(100),sei(100),sem(100),sen(100),seo(100)*
	CC20	*hla(100),hlg2(100),icaA((100),clfA(100),sdrC(100),sdrD(100),sdrE(100),lukE(100)*	*seg(100),sei(100),sem(100),sen(100),seo(100)*
	CC15	*pvl(100),hla(100),hlg2(100),icaA(100),clfA(100),sdrC(100),sdrD(100),lukE(100)*	*sed(100)*
	CC30	*hla(100),hlb(100),hlg(100),icaA((100),clfA(100),sdrC(100),lukE(100),tsst(100)*	*sea(100),seg(100),sei(100),sem(100),sen(100),seo(100)*
	CC22	*pvl(100),hla(100),hlg(100),icaA((100),clfA(100),sdrD(100),sdrE(100),,lukE(100)*	*seg(100),sei(100),sem(100),sen(100),seo(100)*
	CC25	*hla(100),hlg2(100),icaA(100),clfA(100),sdrC(100),sdrD(100),sdrE(100),bsa(100), lukE(100)*	*seb(100),seg(100),sei(100),sem(100),sen(100),seo(100)*

Adhesion genes were present in most *S. aureus* isolates; 100% carried the *icaA* and *clfA* genes, 85.9% harbored *sdrC*, and 69.2% carried *sdrE*.

The most prevalent toxin-encoding genes detected were *hla* (100%), *lukE* (89.8%), *hlg*2 (84.6%), and *hlb* (57.7%). The positivity rates for *tsst1* (5.1%) and *eta* (3.8%) among all BSI isolates were low. The *pvl* gene was detected in 14 strains, which represented six different STs, with ST59 being the most common. The presence of staphylococcal enterotoxin genes was strongly associated with the MLST profile. Thirteen classical enterotoxin genes (*sea*, *seb*, *sec*, *sed*, *see*, *seg*, *seh*, *sei*, *sem*, *sen*, *seo*, *seq*, and *sek*) were detected within the 78 BSI strains (**Table 2**). Overall, each enterotoxin gene was found in multiple *S. aureus* isolates, ranging from 5.1% to 23.1%. No enterotoxin gene was found in ST398 and ST630 isolates. *see*-*sep* genes were present in ST7 and ST88 strains, whereas *sed*-*sej* genes were present in ST5 and ST487 strains. All ST5, ST20, ST22, ST25, ST26, ST30, ST121, and ST487 strains harbored *seg*-*sei*-*sem*-*sen*-*seo* genes, but all ST59 isolates carried *seb*-*sek*-*seq* genes.

### Molecular Characteristics of MSSA and MRSA

Both MSSA and MRSA showed considerable genetic diversity. Fourteen distinct STs and 31 *spa* types were identified among MSSA isolates, whereas nine STs and 15 *spa* types were found with MRSA strains. Among the MSSA isolates, ST7-MSSA and ST188-MSSA (14.1% each) represented the most predominant clone, followed by ST5-MSSA (7.7%) and ST6-MSSA (6.4%). Among MRSA isolates, ST59-MRSA- SCC*mec*IV/V (15.4%), the predominant CA-MRSA clone in China, still represented the most common clone. ST398, ST88, ST188, ST1, and ST30 were identified among both MSSA and MRSA isolates.

All MRSA isolates were resistant to oxacillin, penicillin, and cefoxitin. All MSSA isolates were susceptible to cefoxitin, oxacillin, moxifloxacin, levofloxacin, and rifampicin. In general, MRSA strains showed much higher resistance rates to the tested antibiotics than MSSA strains except in the cases of ciprofloxacin, trimethoprim-sulfamethoxazole, and gentamicin (**Table 3**).

**Table 3:** Antimicrobial susceptibility profiles of methicillin-susceptible *Staphylococcus aureus* (MSSA) and methicillin-resistant *S. aureus* (MRSA) isolates.

	*S. aureus* (*n* = 78), R^a^ (%)	MRSA (*n* = 27), R^a^ (%)	MSSA (*n* = 51), R^a^ (%)	*P*-value^b^
FOX	34.6	100	0	< 0.05
LZD	0	0	0	
CIP	5.1	3.7	5.9	> 0.05
DA	44.9	63	35.3	< 0.05
E	46.2	63	37.3	< 0.05
SXT	9	3.7	11.8	> 0.05
MOF	1.3	3.7	0	> 0.05
V	0	0	0	
TET	23.1	29.6	19.6	> 0.05
P	93.6	100	90.2	> 0.05
RD	1.3	3.7	0	> 0.05
LEV	2.6	7.4	0	< 0.05
OXA	34.6	100	0	< 0.05
GM	3.8	3.7	3.9	> 0.05
Q/D	0	0	0	

^a^*R = resistance. ^*b*^The resistance rates of antimicrobials among MRSA strains were compared to those among MSSA isolates. Cefoxitin (FOX), linezolid (LZD), ciprofloxacin (CIP), clindamycin (DA), erythromycin (E), trimethoprim-sulfamethoxazole (SXT), moxifloxacin (MOF), vancomycin (V), tetracycline (TET), penicillin (P), rifampicin (RF), levofloxacin (LVX), oxacillin (OXA), gentamicin (GM), quinupristin/dalfopristin (Q/D), and tigecycline (TGC)*.

All isolates harbored *icaA*, *clfA*, and *hla* genes. Fifty (64.1%, 50/78) isolates, including 31 (60.8%) MSSA and 19 (70.4%) MRSA strains, harbored ≥ 10 of the tested virulence genes evaluated in this study. The positivity rates for *pvl* among MRSA and MSSA isolates were 37.0% (10/27) and 7.8% (4/51), respectively. The virulence genes *pvl*, *hlb*, *sdrC*, *lukE*, *tsst*, *seb*, *sek*, and *seq* were significantly more common in MRSA strains, whereas only *sdrE* was more prevalent in MSSA strains (**Table 4**). MSSA isolates consistently lacked *sek* and *seq*, and these genes were only present in MRSA isolates.

**Table 4:** Frequencies of virulence genes among methicillin-susceptible *Staphylococcus aureus* (MSSA) and methicillin-resistant *S. aureus* (MRSA) isolates.

Virulence gene	*S. aureus* (*n* = 78)	MRSA (*n* = 27)	MSSA (*n* = 51)	*P*-value^a^
*pvl*	17.9	37.0	7.8	< 0.05
*hla*	100	100	100	
*hlb*	57.7	88.9	41.2	< 0.05
*hlg*	15.4	22.2	11.8	> 0.05
*hlg2*	84.6	81.5	86.3	> 0.05
*bsa*	17.9	14.8	19.6	> 0.05
*icaA*	100	100	100	
*clfA*	100	100	100	
*sdrC*	85.9	100	78.4	< 0.05
*sdrD*	50	29.6	60.8	< 0.05
*sdrE*	69.2	66.7	70.6	> 0.05
*lukE*	89.8	77.8	96.1	< 0.05
*lukM*	0	0	0	
*arcA*	0	0	0	
*tsst1*	5.1	11.1	2.0	< 0.05
*eta*	3.8	0	5.9	> 0.05
*etb*	0	0	0	
*sea*	21.8	25.9	19.6	> 0.05
*seb*	23.1	55.6	5.9	< 0.05
*sec*	17.9	22.2	15.7	> 0.05
*sed*	12.8	7.4	15.7	> 0.05
*see*	20.5	14.8	23.5	> 0.05
*seg*	20.5	7.4	27.5	> 0.05
*seh*	5.1	3.7	5.9	> 0.05
*sei*	23.1	11.1	29.4	> 0.05
*sej*	6.4	0	9.8	> 0.05
*sek*	19.2	55.6	0	< 0.05
*seq*	19.2	55.6	0	< 0.05
*sel*	5.1	7.4	3.9	> 0.05
*sem*	21.8	11.1	27.5	> 0.05
*sen*	21.8	11.1	27.5	> 0.05
*seo*	21.8	11.1	27.5	> 0.05
*sep*	20.5	14.8	23.5	> 0.05

^a^*The positive rates of virulence genes among MRSA strains were compared to those among MSSA isolates*.

## Discussion

Recently, the incidence of community-associated MRSA infection has been increasing. CA-MRSA typically presents as skin and soft tissue infection (SSTI), but invasive infection such as bacteremia can occur, which can lead to serious or even fatal consequences, especially in children and immunocompromised patients. Given this dangerous consequence of CA-MRSA infection in pediatric patients, there is an urgent need to understand the prevalence, molecular characteristics, and virulence profiles of CA-MRSA strains isolated from BSIs to initiate measure to control infection and transmission in local communities.

The occurrence rate of CA-MRSA varies substantially worldwide, ranging from < 1% to > 50% in different countries (Deurenberg and Stobberingh, 2008; Chen and Huang, 2014). In an ANSORP study conducted in 17 hospitals in eight Asian countries, namely Korea, Taiwan, Hong Kong, Thailand, the Philippines, Vietnam, India, and Sri Lanka, the rate of MRSA among CA-SA infections ranged from 2.5 to 39% (Chen and Huang, 2014). In addition, the rate of CA-MRSA among childhood infections in Taiwan increased significantly from 9.8% in 1999–2000 to 56% in 2004–2005. In China, based on a study performed in Beijing Children’s Hospital, 14(4%) of 351 CA-SA infections were caused by MRSA (Wu et al., 2010). These studies focused on CA-MRSA infection, mainly from SSTIs. Few data describing the rate of CA-MRSA isolated from BSI are available. As a result, we characterized 78 CA-SA isolates recovered from pediatric patients with BSIs between 2012 and 2017 in Shanghai and identified 34.6% as MRSA strains. This indicates that MRSA is an important pathogenic bacterium associated with severe, life-threatening infections in children, and suggests the urgent need for active surveillance of such infections and transmission in children.

Community-associated-methicillin-resistant *S. aureus* infections have been reported globally and the major pandemic clones are frequently related to specific geographic areas (Mediavilla et al., 2012; Li et al., 2016). clones with ST59 are mostly found in the Asia-Pacific region including Taiwan and Australia (Chen and Huang, 2014). In China, ST59-SCC*mec*IV/V-t437 is always identified as the major epidemic CA-MRSA clone (Geng et al., 2010). Our current data confirms previous findings with respect to the prevalence of ST59, but minor differences were noted. ST59-SCC*mec*IV-t172 was present with increasing prevalence and became the most common CA-MRSA clone in Shanghai. Although there is considerable genetic diversity among MSSA clones observed in China, ST188 and ST7 have been reported to be the most prevalent clones from adult BSI cases. Our result was consistent with these data, with these strains accounting for 29.5% of all isolates. Recently, ST188 and ST7 were identified as the predominant types among MSSA strains from childhood pneumonia between January 2014 and June 2015 (Song et al., 2017). Furthermore, Our previous study also found these were the common types that caused bovine mastitis from 2014 to 2015 in Shanghai and Zhejiang areas (Li et al., 2017), implying that among the species, some prevailing clones have arisen and spread throughout China.

In the current study, ST398, ST188, ST88, and ST1 were similarly identified among both MSSA and MRSA strains, which suggested that these MSSA lineages probably provide a stable genetic environment for the integration of SCC*mec* to facilitate MRSA infection and transmission in both healthcare facilities and communities. Of note, ST398 was originally found to be a MSSA strain in China, and increasing numbers of ST398-MRSA isolates have emerged in many regions. Historically, ST398-MRSA, first referred to as livestock-associated MRSA (LA-MRSA), has been reported among pigs and pig farmers in France and the Netherlands (Graveland et al., 2011; Fluit, 2012). After, it has become the overwhelmingly dominant lineage in Europe and North America. In the current study, we identified nine ST398 isolates (**Tables 3**, **4**) including five MRSA and four MSSA strains. It is very difficult to speculate on the origins of these isolates because of the absence of epidemiological data linking these to animals. However, LA-SA usually harbored an intact beta-toxin gene (*hlb*) and no lysogenic prophages encoding the immune evasion complex genes (*sea*, *sep*, *sak*, *scn*, and *chp* genes) (van Wamel et al., 2006). Among ST398 isolates in our study, they all had an intact *hlb* gene and didn’t contain *sea* and *sep* genes. Three strains lacked all the immune evasion complex genes, and others harbored one, two or three of *sak*, *scn* and *chp* genes. This was important evidence that these strains were of animal origin. Therefore, it is very important to carefully monitor the animal-to-human transmissibility of LA-SA.

The pathogenicity for *S. aureus* mainly relies on the presence of a variety of virulence factors that mediate adhesion, invasion, persistence, tissue invasion, the evasion/destruction of host defenses, and toxin-related disease (Dinges et al., 2000; Wardenburg et al., 2007; Diep and Otto, 2008). These include microbial surface protein (Josefsson et al., 1998), hemolysin, toxic shock syndrome toxin-1, exfoliative toxins, and SEs. In this study, adhesion genes were present in most *S. aureus* isolates; 100% carried the *icaA* and *clfA* genes, 85.9% harbored *sdrC*, and 69.2% carried *sdrE*. The high prevalence of adhesion genes in clinical strains is consistent with the notion that the adherence of *S. aureus* to host cells is the first vital step for bacterial pathogenicity. The presence of *pvl* has previously been strongly associated with CA-MRSA infections in many studies (Lina et al., 1999). We found ten MRSA isolates and four MSSA isolates that harbored *pvl*. The prevalence of *pvl* in MRSA isolates (37.0%) was significantly higher than that in MSSA isolates (7.8%), supporting the hypothesis that *pvl* is a possible marker for CA-MRSA. In addition, the distribution of some virulence genes, especially SEs, was reported to be closely associated with specific molecular types (He et al., 2013; Wang et al., 2016). A similar result was observed in the current study. The *seb*-*sek*-*seq* genes were present in all ST59 strains, whereas the *seg*-*sei*-*sem*-*sen*-*seo* genes were found to be present in different clones, including ST5, ST20, ST22, ST25, ST26, ST30, ST121, and ST487 strains. In addition, ST5 and ST59 isolates harbored more enterotoxin-encoding genes than ST188 and ST398 strains, implying that different molecular types are associated with different virulence profiles.

The current work has several limitations. Most importantly, the relatively small sample size from a single center limited the representative significance of the research. Second, it is not known whether different genotypes are associated with clinical outcomes because of the absence of clinical information. In addition, as retrospective research, it is possible that patients were misclassified as CA due to incomplete data. However, this study revealed that community-associated *S. aureus* strains from BSIs in children demonstrated considerable genetic diversity and identified major genotypes of CA-MRSA and CA-MSSA, with a high prevalence of MRSA.

## Author Contributions

ML and XW designed the studies and obtained funding. XW, QL, HZ, WH, and QF performed the experiments and/or analyzed the data. ML, XW, and XL wrote the manuscript.

## Conflict of Interest Statement

The authors declare that the research was conducted in the absence of any commercial or financial relationships that could be construed as a potential conflict of interest.

## Footnotes

1 http://saureus.mlst.net

2 http://www.ridom.de/spa-server/
